# Assessing DNA rates within first time psychiatric referrals and the extent to which DNA rates are reduced by an SMS reminder service

**DOI:** 10.1192/bjo.2021.831

**Published:** 2021-06-18

**Authors:** Henry Coates

**Affiliations:** University of Birmingham

## Abstract

**Aims:**

1) To assess the average wait time for patients to be offered an appointment and to establish any correlations between longer waiting times and 'Did not attend (DNA)' rates 2) To assess the number of patients who have opted into the text message appointment reminder service and whether this had an effect on DNA rates.

**Background:**

Research has indicated that the Did Not Attend (DNA) rate in Psychiatry is estimated at 20%, twice that of other medical specialties (1). With NHS Digital estimating that DNAs cost the NHS £1 Billion per annum, there has been much interest in reducing the rate of DNAs within Psychiatry (2). Findings have shown that short waiting times are associated with higher rates of attendance (3). In addition, poor appointment attendance within Psychiatry is also associated with increased disease severity and higher rates of hospital admission (4).

**Method:**

We conducted retrospective data collection on 99 patients referred to Professor Oyebode between January 2018 and August 2019. Our data collection involved assessing time the referral was received, time to first appointment and the patient's communication preference (e.g. whether they opted in to the SMS alert service). All data collection was conducted through use of RIO and coded/ammonized into a Excel spreadsheet. No sampling methods were employed and our population only consisted of first-time referrals to Professor Oyebodes clinic.

**Result:**

1) We found no correlation between a longer waiting time to first appointment and an increased DNA rate.

2) All patient waiting times between 1st January - 31st August were within the maximum limit set by national guidelines

3) Opting into the text messaging service remains severely low. Of the patients audited, 95% had not completed a communication preference form. Overall, it is still unclear whether the text messaging service has a positive impact on DNA rates.

**Conclusion:**

Our data have shown no significant correlation between a longer waiting time and an increased DNA rate for first time Psychiatry appointments. Secondly, we have concluded that between the audited period, waiting times were still within the maximum 18 week wait set by the Mental Health Standards. Finally, we can conclude that uptake of the text messaging service remains very low at 4%. Due to a limited sample size of only 4 patients, it is still unclear from this audit whether opting into the text messaging services will have a positive decrease on the number of DNA's.

